# The Quest for Meaning Around Self-Injurious and Suicidal Acts: A Qualitative Study Among Adolescent Girls

**DOI:** 10.3389/fpsyt.2019.00190

**Published:** 2019-04-05

**Authors:** Salomé Grandclerc, Solene Spiers, Michel Spodenkiewicz, Marie Rose Moro, Jonathan Lachal

**Affiliations:** ^1^Maison de Solenn, MDA Cochin, AP-HP, Paris, France; ^2^Université Paris Descartes, Sorbonne Paris Cité, Paris, France; ^3^CESP, Fac. de Médecine - Univ. Paris-Sud, Fac. de Médecine - UVSQ, INSERM, Université Paris-Saclay, Villejuif, France; ^4^Service de Pédopsychiatrie, CHU de Caen, Caen, France; ^5^UFR de Médecine, Université Caen Normandie, Caen, France; ^6^Université Paris 13, Villetanneuse, France; ^7^Pôle de Santé Mentale, CHU Sud Réunion, Saint-Pierre, France; ^8^CEPOI EA 7388, UFR Santé, Université de la Réunion, Saint-Pierre, France

**Keywords:** NSSI, suicidal behavior, suicide, self-injury, adolescent, qualitative study, experience

## Abstract

**Introduction:** Suicide and non-suicidal self-injury (NSSI) are major problems in adolescent psychiatry and share numerous clinical characteristics. The principal objective of this study is to describe the subjective experience of adolescent girls and young women who present NSSI and/or suicidal behaviors and to determine the common aspects and the specificities of each experience.

**Method:** This exploratory study is based on a procedure that is qualitative, phenomenological, and inductive. The data were collected from two semi-structured interviews each of 18 girls and young women aged 12–21 years, who were receiving care from a psychiatrist specializing in adolescents and who at least once had harmed themselves by NSSI or attempted suicide, or both. The thematic data analysis was performed by applying the methods of interpretative phenomenological analysis.

**Results:** The results are described around four superordinate themes: relationships with the self, with others/otherness, with the body, and with death; they are then subdivided into 13 themes. Several themes appeared common to both types of behavior, especially the relational dimension of self-harming acts. The process of separation-individuation seems complex for these youth.

**Conclusion:** The results underline the relational aspects of the self-harming behavior (NSSI or suicidal) among adolescent girls. These aspects also appear to be expressed in the family sphere, the social sphere, in contact with peers, and also at a societal level when the community no longer addresses adolescents' difficulties. When the process of subjectification does not appear to reach completion, self-aggressive behavior is seen as an ultimate attempt to regain a feeling of autonomy.

## Introduction

Suicide and nonsuicidal self-injury (NSSI) are major public health problems in adolescent psychiatry. Worldwide, the prevalence of NSSI is 17.2% in adolescents, 13.4% in young adults, and 5.5% in adults (1–7). The principal risks of NSSI involve its potential to develop either into chronic behavior or toward other forms of self-harming behavior, in particular, suicide attempts (3). Suicide is the second leading cause of death among people aged 15–29 years, and suicide attempts are 10–20 times more frequent than deaths by suicide (8). Adolescence is a period of great vulnerability for both NSSI and attempted suicide (9), especially among young girls, who start these behaviors earlier and perform them at a higher rate than boys (1, 3, 9, 10).

NSSI and attempted suicide share numerous clinical characteristics (11, 12). Some authors treat NSSI as an important risk factor predictive of concomitant or subsequent suicidal behavior or ideation, considering NSSI and fatal suicides as two ends of the same spectrum, two different manifestations of the same behavior (13, 14). It seems impossible to differentiate them at the moment of the act, for in either case, the action, because it may express their inability to imagine death. The act can be considered to short-circuit thought, or perhaps more precisely, intentionality (15). It is only afterwards that the adolescent gives it a meaning.

Young people attribute to NSSI functions of coping, relieving unbearable emotions, and reducing internal tension (16–19). NSSI also appears to create the illusion of regaining control of what is experienced as uncontrollable and menacing or of regaining a sense of reality (16, 20–26). It can make suffering tangible by creating a physical manifestation of a psychic pain difficult to represent mentally (16, 20–34). Young women with previous NSSI describe moments in which they are incapable of recognizing and assimilating their own sensations, of linking their physical and psychological experiences. Emotion remains trapped in the body, unable to connect to a mental representation. This loss of emotional resonance also affects the way they experience reality (30). To perceive blood by seeing and touching it through NSSI, for example, by cutting, may play an important role in their emotional processes (35). In introducing a modification perceived physically, NSSI might allow them to reclaim their emotional experience and their sensory consciousness of themselves (30, 35).

A suicidal act may result more from a dynamic, developmental and evolving process (14, 36–38); the relational insecurity that is evidence of early painful experiences is frequently found in suicidal adolescents (14, 36–45). Their inability to cope with the emotions raised by the discovery of new objects reminds them of the insecurity of their first relationship. The fragility of their ways of relating to others hampers the strategies they use to regulate distress (36, 40, 46–50). Young people, whose attachment bonds as infants had been poor may be more prone in adolescence to apply maladaptive coping strategies in the face of difficult relational experiences (40, 51). They thus have the impression that they have no control over either their internal world or their environment, and the suicidal act appears to them to be a means of regaining control of their own existence (48, 49, 52, 53).

Suicide attempts and NSSI may thus share some of the same objectives—to regain control of their own experience, whether it is internal, physical, or relational. The literature includes clear descriptions by adolescents of this search for control through suicidal or self-harming acts (16, 48). Although these two behaviors frequently occur in the same individuals (54, 55), they are rarely studied together, as we will do in this study, while simultaneously taking their potential differences into account (56).

The principal objective of this study is to describe the subjective experience of adolescent girls and young women who present NSSI and/or suicidal behaviors to determine the common aspects and the specificities of each experience, specifically the meanings that these youth attribute to the self-injurious behaviors that they have previously performed. The secondary objectives are to gain a better understanding of the mechanisms leading to acts of NSSI and suicidal behaviors and to study in detail the relationships they have with those they are close to.

## Materials and Methods

This exploratory study applies a qualitative, phenomenological, and inductive process. Qualitative methods are particularly appropriate for the complexity of the topic we study here, aiming to describe and understand in depth the phenomena observed (57, 58). Qualitative research makes it possible to study the relations between several factors, to take the impact of context into consideration, especially social context. It thus appears complementary to quantitative research in suicide-related topics (59–61), by exploring both suicidal behavior and self-injury in their individual, environmental and socio-cultural complexity. Both context and complexity are especially important in describing subjective experiences. The central role it assigns to empirical results means that the inductive procedure enables the appearance of original findings (57). Finally, the phenomenological framework appears most appropriate for studying the experience of distress. It is very close to the approach used in clinical psychiatry (62–64).

### Sampling

Sampling was purposive, and the subjects selected were representative of typical cases (65). We chose to recruit a population of girls and young women. The prevalence of acts of both suicidal and NSSI is clearly highest among them (1, 9, 10, 66). Moreover, in both the literature (9, 42, 44, 45, 67) and our clinical experience, the experience of young women and young men appear very different and therefore must be studied separately, at least initially.

The girls and young women were recruited within the Maison de Solenn (Hospital Cochin, AP-HP, Paris, France) and in the department of child and adolescent psychiatry of the Caen Normandy UHC (Caen, France). All were aged 12–21 years old during the study period. All must have at least once intentionally committed an act of aggression against themselves (NSSI or attempted suicide). An acute delusional state was an exclusion criterion.

### Data Collection

Research team members suggested face-to-face to girls and young women that they thought might want to participate in this study and organized an informational meeting in the parents' presence before obtaining written consent from both the adolescents and their parents.

A researcher, following an interview guide specifically designed by the research team for this study (Table 1), met at the hospital with each youth alone, twice, in semi-structured interviews of around an hour each.

**Table 1:** Interview guide.

**RESEARCH INTERVIEW n°1**
- Could you explain to me why, in your opinion, you are receiving care in this department?
- Could you tell me what led you to hurt yourself, in your opinion?
- In what circumstances did these acts take place? Can you tell me more?
- How are things in your family? With your parents? Your brothers and sisters? How do you get along? Are you similar?
- How are things at school? With your friends?
- For the next interview we have, I would like to ask you to bring an object that's associated with your action. It could, for example, be a concrete object, a text, an image, or even a song, but it must be directly related to your act. At our next appointment, we will discuss this object, its history, and what it represents to you.
**RESEARCH INTERVIEW n°2**
- Can you tell me why you chose this object, text, image, or song to remember your action? How does it represent that for you?
- What memories are associated with it?
- How do you see the fact of having injured yourself? How do you see the fact of having attempted suicide? What sense does it make for you?
- What are its reasons, in your opinion?
- How do you think your family experienced this act (or these acts)? Do you have the feeling that your relationships had changed, either before or after?

*This table summarizes the “starter” questions used to guide the two research interviews with each of the adolescents*.

During the first interview, the researcher explored the girls' narratives of their life history, their self-injurious or suicidal action, and their relationships with family and friends. The researcher then suggested that the youth think about and choose an object (a physical thing, or something written, or an image, or a song) associated with her action. This is a technique of visual narrative (68). The object served as a basis for the second interview. It was not itself the object of the analysis and interpretation; rather it helped them to talk freely and aided in the co-construction of meaning.

The second interview thus focused around the choice of the object. The girl or young woman was asked to describe it exactly, to explain why she chose it, and to describe the memories associated with it. The different themes discussed in the first interview were considered in greater depth via the narrative around the object. New themes could also emerge.

Two researchers (SG and SS) conducted the interviews. All interviews were audio-recorded and transcribed literally and in full, including the questions after the interview.

### Ethical Standards

This study was carried out in accordance with the recommendations of an appropriate ethics review board (CEERB Paris Nord, IRB 00006477) with written informed consent from all subjects. All subjects (adolescents and their parents) gave written informed consent for the research and for the publication of the datasets (social and demographic characteristics of the study population and direct quotes from the participants) in accordance with the Declaration of Helsinki. The protocol was approved by the ethics review board CEERB Paris Nord. All interviews have been anonymized to get the datasets non identifiable.

### Analysis of Results

The analysis applied the method of Interpretative Phenomenological Analysis (IPA), which makes it possible to study the meaning subjects construct from their experiences (49, 53, 69, 70). Meticulous analysis of the interviews as they were completed enabled us to identify a set of superordinate themes, each linked to several themes describing all of the experiences narrated. The themes were not chosen solely on the basis of their prevalence in the data. Other factors, including the richness of the passages showing the themes and the ways they illuminated other aspects of the narrative were also considered. Two researchers (SG and JL) analyzed the interviews to optimize the validity of the results.

The methodological criteria were retrospectively verified according to the COREQ (COnsolidated criteria for Reporting Qualitative research) checklist (Data Sheet 1).

## Results

### Population

The study included 18 girls and young women between August 2015 and December 2017. All adolescents and their parents gave their informed consent before inclusion. Their characteristics are summarized in Table 2. They comprise a clinical population with fairly severe but nonetheless varied symptoms. For reasons connected to our phenomenological method, we do not report the psychiatric diagnoses assigned to each participant, but some interviews provide clinical information related to the course of care. The NSSIs mainly involved self-cutting or abrasions by repeated scratching or friction. The suicide attempts were principally by intentional self-poisoning, mainly drug (medication) overdoses, or by suicidal self-harm. Of the 18 youth, 16 had been or were then performing chronic self-harming behavior, and no improvement had been seen for 5 of them for several months. The sample was heterogeneous in its socioeconomic status.

**Table 2:** Characteristics of the study population.

**Sex**	**N^**°**^**	**Age at interview**	**Country of origin**	**Psychiatric history**	**NSSI**	**Suicide Attempt (SA)**	**School**	**Current disorder**
F	1	18	France	- History of one hospitalization - Psychological treatment	- Age of onset: 16 - Type: self-cutting - Chronic - Severe injuries - Atypical presentation: abdomen, chest	- One impulsive SA - By medication overdose - High risk of lethality	- In urban school - Year 12	Anorexia nervosa Bulimia Borderline personality disorder
F	2	16	France	- History of two hospitalizations - Previous total disconnection from school - Psychological treatment	- Age of onset: 15 - Type: self-cutting, friction - Chronic	None	- Not - currently attending school - Year 11	Anxiety-based school refusal Anxiety Phobia
F	3	16	France	- History of two hospitalizations - Day hospital - Previous total disconnection from school - Psychological treatment	- Age of onset: 14 - Type: self-cutting - Chronic - Severe injuries	- Several impulsive SA - By medication overdose or NSSI with a suicidal aim	- In urban school - Year 11	Severe depressive episode without psychotic symptoms Anxiety Phobia
F	4	17	France	- History of one hospitalization - Pharmacological and psychological treatment	- Age of onset: 16 - Type: self-cutting - Chronic	- Two planned SA - By medication overdose	- In urban school - Year 11	Severe depressive episode without psychotic symptoms
F	5	21	France and Turkey	- History of anorexia nervosa and bulimia - Psychological treatment	- Age of onset: 16 - Type: self-cutting - Chronic - Severe injuries - Stopped 2 years earlier	None	In school Second year under-graduate	Borderline personality disorder
F	6	14	France	- History of anorexia nervosa and bulimia - History of one hospitalization - Psychological treatment	- Age of onset: 11 - Type: self-cutting - Chronic - Severe injuries - Atypical presentation: abdomen, chest	- Two impulsive SA - By medication overdose or NSSI with a suicidal aim - High risk of lethality	- In urban school - Year 9	Severe depressive episode without psychotic symptoms
F	7	19	France	- History of two hospitalizations - Previous total disconnection from school - Psychological treatment	- Age of onset: 16 - Type: self-cutting - Stopped several months earlier	None	- In school by distance learning - Year 12	Severe depressive episode without psychotic symptom Anxiety-based school refusal
F	8	17	France	- History of one hospitalization - Day hospital - Previous total disconnection from school - Pharmacological and psychological treatment	- Age of onset: 15 - Type: self-cutting - Chronic - Severe injuries - Atypical presentation: abdomen - Stopped one year earlier	- Two impulsive SA and one planned SA - By medication overdose or NSSI with a suicidal aim - High risk of lethality	- In urban school - Year 11	Severe depressive episode without psychotic symptoms Anxiety-based school refusal
F	9	15	France and Senegal	- History of one hospitalization - Day hospital - Previous total disconnection from school - Pharmacological and psychological treatment	- Age of onset: 13 - Type: scratching or friction - Chronic	- One impulsive SA - By medication overdose	- Not attending school - Year 9	Severe depressive episode without psychotic symptoms
F	10	13	France and Serbia	- History of three hospitalizations - Pharmacological and psychological treatment	- Age of onset: 12 - Type: scratching or self-cutting - Chronic	- Three planned SA - By medication overdose	- In urban school - Year 7	Severe depressive episode without psychotic symptoms
F	11	16	France and Morocco	- History of two hospitalizations - Previous total disconnection from school - Psychological treatment	- Age of onset: 14 - Type: self-cutting - Chronic - Severe injuries	None	- Not attending school - Year 10	Atypical anorexia nervosa Severe depressive episode without psychotic symptoms
F	12	16	France	- History of two hospitalizations - Pharmacological and psychological treatment	- Age of onset: 15 - Type: self-cutting - Chronic - Severe injuries	- Two planned SA in childhood - By hanging	- In urban school - Year 11	Severe depressive episode without psychotic symptoms
F	13	17	France	- History of one hospitalization - Pharmacological and psychological treatment	- Age of onset: 16 - Type: self-cutting - Stopped several months earlier	- Two planned SA - By medication overdose - High risk of lethality	- In urban school - Year 12	Severe depressive episode without psychotic symptoms
F	14	18	France and Argentina	- History of five hospitalizations - Previous total disconnection from school - Pharmacological and psychological treatment	- Age of onset: 15 - Type: self-cutting - Chronic - Severe injuries - Atypical presentation: abdomen	- Two impulsive SA - By medication overdose or NSSI with a suicidal aim	- In school - First year under-graduate	Anorexia nervosa Bulimia Borderline personality disorder
F	15	18	France	- History of seven hospitalizations - Previous total disconnection from school - History of anorexia nervosa and bulimia - Pharmacological and psychological treatment	- Age of onset: 15 - Type: self-cutting - Chronic - Severe injuries - Atypical presentation: face, abdomen, chest	- Six impulsive SA - By medication overdose - High risk of lethality	- Not attending school - Year 12	Borderline personality disorder Substance use disorder
F	16	16	France	- History of two hospitalizations - Pharmacological and psychological treatment	- Age of onset: 15 - Type: self-cutting - Chronic - Severe injuries - Stopped several months earlier	None	- In urban school - Year 12	Severe depressive episode without psychotic symptoms Anorexia nervosa Bulimia
F	17	14	France	- History of two hospitalizations - Psychological treatment	- Age of onset: 12 - Type: self-cutting - Chronic - Severe injuries	- Two planned SA - By medication overdose	- In urban school - Year 9	Severe depressive episode without psychotic symptoms Anorexia nervosa
F	18	16	France	- History of one hospitalization - Previous total disconnection from school	- Age of onset: 14 - Type: self-cutting - Chronic - Severe injuries	None	- Not attending school - Year 11	Severe depressive episode without psychotic symptoms Anorexia nervosa

*This table summarizes the social and demographic characteristics of the study population. It also mentions their psychiatric history, their current disorder, and the type of act*.

### Study of Objects

Of the 18 young people included, 12 followed the instructions to bring an object associated with their act to the second interview. These objects provided the subjects with a way to tell their story supported by the memories associated with it. The choices of object varied. It did not systematically allow them to generate a discourse on our research topic, but it nonetheless shed an interesting light on their relation to their acts and on their representations. Table 3 identifies the different types of objects, the meanings attributed to them, and the themes associated with them.

**Table 3:** Study of objects.

**Participants**	**Object reminding them of the act**	**Meaning attributed to the object**	**Related themes**
Girl 1	**Food** Systematically performs NSSI after bulimic episode	Hatred of food, hatred of self.	***Inner combat*** Symbolized by her relation with food.
Girl 2	**No object**	Said she had “no idea”, “did not know what to bring.”	***The body, which makes it possible toregain control of the emotions*** Difficulties in representing the act to herself.
Girl 3	**Song: “Somewhere only we know”** **performed by Keane** Listened to it regularly when she cut herself	Song chosen with her boyfriend to remind each of the other.	***Search for the other's reaction*** Act used as a language.
Girl 4	**Tweezers** First object used to cut herself	Object within easy reach, not causing severe cuts.	***The action as a moment alonewith oneself*** Moment of privacy/intimacy.
Girl 5	**Two songs: “Skin” by Sixx: A.M., “Scars”by Papa Roach and a poem by Baudelaire** **“L'héautontimorouménos” (“The Self-Tormentor”)** Recalling the previous period of distress and self-cutting	Search for the meaning of her pain and her need to hurt herself through the lyrics of these songs and this poem.	***NSSI that isolates and connects*** Experience of not belonging and search for a group capable of understanding her message.
Girl 6	**Three texts she wrote herself** In moments of distress, often after self-injury	Texts in which she talks of her vulnerability, of her relationships with others, often undermined by her distress.	***The act as a test of the separation processin adolescence*** Process of separation and interdependence.
Girl 7	**Free verse she wrote herself** After a moment of cutting	Text written to raise questions about the relation between her body and her distress.	***Limits of the body and limits of the self*** Teach her body to suffer less.
Girl 8	**“My arm”** “I don't need an object, (…) it's my arm, my object…”	The scars of self-cutting as symbols.	***The body that enables the feelingof existence*** Writes her distress in her flesh.
Girl 9	**Razor blades and medication**	Did not use razor blades to cut herself but she brings one as an object reminding her of cutting and not of her cutting.	***NSSI that isolates and connects*** Affiliation creating a “cultural” belonging.
Girl 10	**Bracelet given to her by her cousin livingin the West Indies**	The bracelet represents her self-injurious acts and at the same time a comforting object reminding her of her family in a faraway country.	***Inner combat*** The object symbolizes the double valence of the act.
Girl 11	**No object**	She can't explain why she did not choose an object.	
Girl 12	**A white sheet of paper with ink on it, but no words**	Association with the fact that a sheet of paper can cut and that ink calls blood to mind. Could not explain this very figurative representation.	***The body, which makes it possible toregain control of the emotions*** Words are not used to “fill in” this white sheet but the ink is deposited, without form.
Girl 13	**Scissors, her grandfather's pill box, places** **where she's thought of suicide attempts**	Object used for her first cutting.Recollections where she thought of suicide/suicidal plans.	***Acting, alone on stage*** Acts revisited as scenes with precise memories of the objects and places associated with them.
Girl 14	**Two songs: “Breathe me” by Sia and** **“Ta meilleure amie” (Your best friend) byOrnella Tempesta**	Recognizes herself in the words of these songs which have in common asking another for help.	***The act: sending a message*** She chose these songs for another to receive, although she denied in the interview the existence of a message behind her NSSI behavior.
Girl 15	**No object**	Did not succeed in finding a metaphor for her acts, but explained that they were necessarily associated with very specific memories, which she was unable to say more about.	
Girl 16	**No object**	Cannot explain why she did not choose an object.	
Girl 17	**No object**	Cannot explain why she did not choose an object.	
Girl 18	**No object**	Cannot explain why she did not choose an object.	

*The table summarizes the girls' responses to the instruction to bring an object associated with their act to consider in light of the themes obtained from the analysis of the interviews*.

### Thematic Analysis

The phenomenological thematic analysis of the interviews enabled us to uncover 13 themes, organized around four superordinate themes: the first concerned their internal (intrapsychic) relationships to the self; the second explored their relationships to others and otherness at an intersubjective level; third, their relations to their body and their senses; and last, their relation to death.

Throughout these interviews, the associations between NSSI and suicide attempts appeared interlinked. NSSI and suicidal experiences may be situated at different levels but were often combined in discourse. We met adolescents who attempted suicide without intending to do so, and others who cut themselves with an ambiguous suicidal intention, in multiple alternating and associated configurations. We have therefore deliberately chosen to present some themes without distinguishing the two behaviors. Nonetheless, we have tried to compare and contrast these two types of experiences and describe how some themes were common to both behaviors or specific to one or the other.

#### Relationship to Self

##### Dependence on self-harm

In the girls' discourse, NSSI frequently took on a function of relief from an emotional state described as intolerable and out of control. Dependence on NSSI can thus develop. Our interviews found the re-establishment of an internal homeostasis in the aftermath of their act, and the increasing feeling of a lack in its absence, as time passed after the act. NSSI appears to serve as a means of resolving an internal experience, of bearing a difficult situation. Dependence develops when it is the only means considered effective and immediately accessible.

Girl 10: “No… Sometimes there are triggers…but… most often not… it's really that … I… I feel a lack and I need to do it [self-injure]… and I do need to in fact … it's more than a triggering factor … it's very rare for there to be a trigger… when I feel bad, uh… I do it…”

Suicidal acts are not part of a situation of build-up and dependence. The girls and young women report less awareness in suicide attempts of the act's immediate feelings and direct consequences. This theme thus appears specific to NSSI.

##### Inner combat

Several interviewees described themselves as in a battle against themselves, in a conflict with no possible negotiation between “two selves” in a situation where one must defeat the other. This result was particularly true for the experience of NSSI.

Girl 1: “Bah, I'm in conflict with myself, I hate myself… I hurt myself but it's as if it's my adversary, but it's me my adversary. I fight against the adversary but the adversary is me… So I'm fighting against myself… So, in this combat, we end up hurting each other… And finally, it's always against myself and…”

This internal conflict can be found in the releasing or purgative function of NSSI, as it becomes a means of getting rid of what is dysfunctioning within oneself and of finding a transient state of peace. Nonetheless, the conflict can never be completely resolved in this manner.

Girl 8: “That's also why I only did it in the shower… There was… there was something dirty inside of me, so, uh … I opened everything so that it could get out, and I washed it with water in fact… It was… a kind of shower in fact…”

Suicide attempts do not have this cathartic function; internal combat is not as accurate a metaphor for them, for in suicidal moments, there is no more battle. The person has reached a level of ill-being such that there is no more possibility of being freed from it, but only of abolishing consciousness.

##### “Another me”: from the experience of possession to the fear of insanity

In these interviews, the girls and young women underlined an experience of strangeness before and during NSSI and suicidal experiences. They described a *longing* to hurt themselves, a *longing* to kill themselves. This longing, almost unthinkable for them, led to two explanations: insanity, or the more acceptable representation that there is *another me* or an alter ego, although this never involved an experience of psychotic splitting. They described a form of possession that results in a loss of control of themselves.

Girl 1: “Instantly, you're possessed.”

Interviewer: “Does that come up often in your discourse–possession?”

Girl 1: “Yeah… But there's someone in me… another me.”

*Girl 17:* “*And I had anxiety attacks, I had the impression I was going crazy. I had the impression that cutting myself, that would bring me back to my senses.”*

She described her act, an integral part of this experience, as a constraint, imposed on her. The act is thus not voluntary and is experienced passively. Acts of self-harm, whether they involve NSSI or suicide, are associated with a feeling of relief, synonymous with a partial resumption of self-control.

Girl 12 (talking about the first time she cut herself): “I was simultaneously conscious and no, not really…I was conscious that I was hurting myself and that I really had wanted to do it, but in some way, I wasn't really in my right mind to do it, I was really in bad shape…Talking wasn't enough.”

##### The action as a moment alone with oneself

The NSSI or suicidal action is presented as a moment of intimacy, belonging to one's secret garden, where the other no longer exists, where one is alone: a moment “alone with myself,” like a secret ceremony performed as an intimate liturgy. The question of pleasure seems present in this theme: an intimate but a painful pleasure.

Girl 16: “I thought that it was a good thing for me. And then even the scars, afterwards I found them pretty, so… (…) I told myself that I was doing myself good. But when my friends saw them, I experienced that very badly because I had nothing left for me.”

These young people mention a selfish need for isolation, without outside interference. But the nature of the process means that this private moment can only rarely remain a secret and can be transmitted within the family sphere, or even more widely in treatment. This theme is common to both types of behavior.

Girl 3: “Yeah… I have my precious…uh… I… I need to be shut away uh … with myself, to not hear the outside world… It's hard for me because… In fact, I would really like to be alone at that moment, I would like no one to know … and… and except at some point they find out… they see it… and… as a result… I… well my parents, they don't handle it well and… well, each time, they call an ambulance…”

#### Relationship to Others/Otherness

##### Search for the other's reaction

The act, be it NSSI or suicidal, seeks to provoke a reaction in another/the other. It is an interactive language calling on the other, and it seems to have a strong relational value. The bond is tested, attacked.

Girl 5: “Yeah, the first time I did it, I remember also… well, uh… I was fighting with my parents and they had broken a frame that really mattered to me and uh… Yeah, I had so much trouble with this frame…with uh… broken things but uh…”

The act is also a mean of controlling the relationship by generating fear, unpredictability, and violence. This gives an impression of frozen interaction, with the family helpless.

Girl 17: “Well, I have the impression that my parents now look at me with a little pity, well, now they are always worried about me, though they weren't before. Even though it was going on in front of them…”

One girl, through a text she wrote and brought to the second interview, illustrated how the act induces an interdependence. The other must always expect the worst. The aim is to always be present in the other person's head.

Girl 6: “You may have figured out that I'm not the good kind of girl. I'm one of those that you can't leave alone on the banks of the Seine, or on its bridges, and still less in buildings. Not too close to knives and keep the meds away. Because you never know what might take me in the black night, what could make me howl after yet another nightmare. I'm one of those you can't leave alone with herself. Because when you come back, you never know what you might find in the bathroom, inert, what you might find all covered with blood in the kitchen.”

##### The act: sending a message

These youth try to communicate a message by their act. This result was found both for NSSI and suicidal acts. The message may be implied, suggested by traces on the body, the words, or the clues left. The first message is to testify to pain and distress. But still more, these girls and young women seemed to expect help from their families in understanding what is happening to them.

Interviewer: “Why would your parents feel guilty?

Girl 16: “well, that they didn't realize right away…because … My mother said ‘I didn't think you would go to that point [to cutting], that you would go so far as to do that… So uh…for not having perceived how bad I've been feeling.”

One young woman did not seem to find any meaning in her cutting and in some ways refused to look for one. This echoed her parents' absence of reaction, their inability to talk to her about this act.

Girl 14: “Well, really, lots of people say that cutting, it's like a call for help, but in my…in me, I never saw it like that because I always hid it… I never wanted anyone to see … and… well, I started to show it precisely when things were getting better…”

Sometimes the relationship was so close that the message did not need to be said, the act itself verified the existence of a metacommunication. The other could understand the hidden message without either needing to speak.

Girl 6: “But I don't talk about it, they see it … They're discovering that I'm … finally they see that I'm not well… finally, you can see when someone is not doing well…they see it and uh… they can't stand it.”

This communication beyond words takes on another meaning when the parents come from elsewhere. The youth may be awaiting her parents' understanding, but the cultural dimension can make it impossible for her to be explicit enough for them to understand.

Interviewer: “And have you have been able to talk about it more calmly yet?”

*Girl 9:* “*No. Well, that is… it's not too much in the culture and then… well, over there… when you do that (NSSI), you're…you're a little…for them, you're crazy…”*

##### The act as a test of the separation process in adolescence

In the interviews, our participants often described their relationships with their mothers as close. The action could be presented, in the girls' discourse, as a violent means of cutting their ties to their mothers or at least of testing the possibility of cutting them, as a metaphor for individuation, for liberating themselves from dependence.

Girl 1: “And nonetheless, after each crisis, I say to my mother: ‘So, you don’t love me anymore, huh? You want to abandon me? You want to throw me out?'. And each time, I say to her, uh: ‘So, have I driven you bananas? So.‘ Uh… So finally I always ask her the same questions. ‘You hate me now, huh?’. She's sick of it…”

Both the NSSI and the suicidal act can be seen as a means of pushing the other away, but also as self-punishment that a girl inflicts on herself for envisioning or desiring separation.

Girl 5: (Talking about NSSI): “On the one hand, I was punishing myself too, because well I was very angry at myself… Because I was… I had a period where I was, uh… I was… well, my mother and I, we were fighting all the time … but really a lot, so my teenage crisis … it was horribly violent and it lasted… And I think that was a way to punish myself for hurting her … then uh… after… after I turned 18, it was more uh… just a way to punish myself … [without it having anything to do with her]”

These movements of coming closer and distancing, mediated by the act, illustrate the work of adolescence. This theme, common to NSSI and suicide attempts, nonetheless has several particularities according to the type of behavior. NSSI can become the object of negotiation around separation.

Girl 11: “They worry too much…. They don't want to leave me alone in the house anymore. My mother, she searches my stuff every time, she always wants to look at my arms, all that…”

Girl 18: “I think that it's not [my parents'] role in fact, to … try to prevent me from cutting myself. I think it's the therapist's role…So I think that they know it, well yes, they know it, but I mean that they shouldn't get involved after, I think …. I think that's not their business in fact…”

A suicidal act can even be the final alternative to separation, in a situation where the youth is unable to envision this process. One girl talked about her mother's depression and suicidal ideation, which she experienced as a threat of abandonment. Her own suicidal gesture can be seen as a means of not risking abandonment and separation again.

*Girl 4: “She cannot be angry at me for these desires because she knows what it is… uh… she's had them too… because well… she, the only thing that held her back, it's my sister and me… after, well me, I don't have a child… so it's a little more complicated…”*.

##### NSSI that isolates and connects

These discourses show substantial difficulties in peer relationships. These young people have often had painful experiences of rejection, lack of understanding, not belonging. These difficulties in developing relationships with peers, in being connected, frequently induce a strong propensity to identify with other youth in distress, and self-cutting is the symbol of distress.

Girl 6: “For me, in fact… to hurt myself, my cutting, etc… it's already a big big part of my life… because it's become my personality, in fact… I was so used to feeling bad that for me now, it's … it's me, in fact. It's my personality, it's… it's me!”

Girl 14: “Because for me, the traces that I have… well the scars I have… it's… it's horrible, but for me, it's going to mean that I will be a stereotype of… of … the troubled adolescent all my life…”

This group bond is found more often for NSSI than for suicidal behavior and is a fundamental difference between them. An illustration of this process is found in their discourse: their use of the term “on” (generally translated into English as an impersonal “one” or as “we” or “you”) when they talk about cutting. This seems to be the starting point of the contagious effects frequently described in NSSI.

Girl 4: “If you haven't experienced it, it's… it's very complicated… Because you're a little in a… in a bubble… a little special. And you can't… we don't manage to make ourselves see reason… We don't manage to …”

The experience of attempted suicide, on the contrary, does not allow connection, or at least not in the same way. The sharing around suicidal acts is often prevented, even hidden, especially since, unlike for NSSI, there are not necessarily visible scars.

#### Their Relation to the Body and to the Senses

This third superordinate theme enables a more specific description of the NSSI experience or at least of the relation to the body of girls or young women who cut themselves. This theme is less significant in the narratives of suicidal experiences; the body is less directly involved, is used less as a means of mediating feelings.

##### The body, which makes it possible to regain control of the emotions

These acts are often associated with states of anxiety, sadness, or anger. The young people in our sample describe themselves as submerged by their emotions. Their difficulty in putting their feelings and emotions into words limits the construction of representations and can thus lead them to act.

Girl 12: (Talking about NSSI): “All of this distress and this suffering…it must have been a shortcut, I think, to do that, I think… It was a little bit the idea in my body that told me: go ahead, do something serious and then like that, you'll get taken care of, someone will take care of you…”

The girls and young women do not succeed in using words and mental representations to distance some feelings or to delay their response to emotions. Emotions are thus experienced physically, painfully, and violently. The act, which is violence to the body, makes it possible to end this pain and regain control of one's body and emotions.

Girl 3: “If there's something that's hurting me… I… I don't have the time in my head in fact to … to… tell myself, ok, calm down! We're going to fix that, all of that…. It's… It's like a pistol in fact… I don't have time to stop it… I don't have time to stop the bullet! So I go off directly… uh… I explode.”

We see extreme sensitivity, even emotional porosity. The emotion is transmitted impalpably, not contained by the meaning of the words.

Girl 7: “Because, uh… I wanted so much to please the others… and I put so much pressure on myself that … I felt really exposed … (…) I was so exposed and disturbed by… by my anxieties, that I had forgotten that I had a protective barrier.”

##### The body that enables the feeling of existence

The act can also help to feel, to perceive this body, in reality, and to learn to deal with its sensations as so much proof of the girl's real existence. In NSSI, cutting themselves and thus being able to visualize, see their blood plays a central role—proves that they are alive. This contributes to the hoped-for relief.

Girl 6: “So, I said to myself that in cutting myself, I would feel pain… And as a result, so… pain and finally I would say: yup, I'm alive … I still exist…”

What is inscribed in the flesh by different forms of NSSI is also engraved in the reality of psychological distress, and this makes it possible to heal it and accordingly to think it. It involves shaping distress that because it is internal cannot be materialized. To see it in flesh and bones engraves it in a reality that can therefore be controlled.

Girl 2: “You have the impression that it's a little more manageable. Because, it disinfects… you tell yourself, it'll pass… it's going to heal. And so, you don't think any more about … about the anguish …”

##### Limits of the body and limits of the self

In their experience of NSSI, the young women are expressing the need to reappropriate their bodies, to set its borders, and to experience the enveloping function of the skin. NSSI can appear to be a carnal experience.

Girl 7: “Because, uh… I felt I was really exposed… I had forgotten that I had a protection in fact … so uh, which was in fact my skin… and I felt, uh… attacked by everyone and I had the impression that everyone could read inside me, that … I was so exposed and disturbed by… by my anxieties, that I had forgotten that I had a protective barrier. And the fact of having hurt myself, that was to remind me that … people couldn't read inside me… and that I was protected from … that I was free of…”

The skin here is a hypersensitive border because the borders between self and the other are difficult to establish; NSSI is a means of denouncing their blurriness, of verifying the permanence of the protective capacity of this physical border, and of creating a material inside-outside interface between self and other.

In other excerpts from the interviews, the traces, marks, the scars of cutting make it possible to control what can be seen, what can be hidden from the other's eyes, thus delimiting the borders of the self and of the body.

Girl 5: “My mother, in fact, she didn't see it… Although there were moments where uh… where I wasn't hiding it … so I realize that … so there it was, I left my arms completely bare… bare… but a moment, so there it was and then I started putting on lots of bracelets too… That irritated her, but uh… she didn't know… And so, on the one hand, I was angry at her for not noticing, and on the other hand, it reassured me, so I was really in a paradoxical situation…”

#### Relation to Death

To different degrees, the themes that will follow are found in the narratives of both NSSI and suicidal experiences. These young people's relations to death raise questions. Death does not always make sense and no longer refers to a concept of irreversibility. It can thus be confused with existence or juxtaposed with it and be asserted as sensation.

##### Testing the lines between life and death

The act is presented as a way of testing the lines between life and death, to take ownership of them. The ambivalence compared with the feeling of immortality of childhood is still present, and youth need to test themselves to learn to deal with the limits of their power and perceive the experience of existence.

Girl 7: “Who makes us doubt this relationship to life? … Generally… so, what, I … what I know, in relation to others… and in relation to myself… is that people hurt themselves to be sure they are still alive. To be sure of it.”

The fear of the uncontrollable nature of death is constant. In the face of it, a suicide attempt appears to be a solution for controlling—for mastering—death.

Girl 12: “You can kill yourself as you like and death is constantly present… and we are destined to die… so, then, I could spend a little time [alive] and at the worst, if I don't like it, I can go… so… it's self-service, so, that's it…”

The narratives report suicidal fantasies behind NSSI. The possibility for NSSI “go wrong” despite one's plans becomes a flirtation with death. The function of the act may be to play an ambiguous, simulated game where reality, pain, and death are all denied.

Girl 8: (Talking about NSSI): “They were a little borderline because finally, uh… each time, I hoped … I hoped to hit a vein in fact… Except… well … I never did…”

##### Acting, alone on stage

The analysis of the results shows that our participants used a form of staging of their action, which raises questions about its exhibitionist dimension. It is described as always taking place in the same place, in the same position, with the same rituals.

Girl 3: “So already, I always do it in my bed… uh… I get into bed, under my quilt, get comfortable…I smoke a cigarette in my bed… and… I put on my music and I take my blade, and I start… And in fact I… And so, like that, … each time I do it while smoking and I try to be as relaxed as possible…”

The concept of *alone on stage* helps to illustrate this experience. It is a staging of oneself in distress, in front of a fantasized public. The youth is alone in this scene, she has the impression of being alone in the middle of others, of being unable to belong to the same group. The fantasies of being watched seem to come into play in the NSSI process but also the suicide attempt: fantasies that the family discovers the razor blade, the traces of blood, the inert body, as a means of existing indelibly in the heads of the others.

Girl 16: “After, I enjoyed it too. So I didn't really want to stop…. There was adrenaline, because well I didn't want my parents to see, so, uh, I did it in the evening, I closed myself in my room, I was always afraid that they'd come back, sometimes they'd come into my room, I had to hide everything in two minutes, the fact of being hidden like that, then also of having a thing that no one knows, having a little secret, it's nice.”

Girl 4: “When I was going to school, I was truly isolated… bah; because there's truly a moment when even though there were people around… you feel very very alone… that means that even, you see people passing by, it's as if … as if they aren't there … (…) And I was like that, and I didn't move for, like an hour, I stayed there with my medication in my hand, … and they went past me, they passed me, and … I was sitting in the hall… no one moved…”

## Discussion

### An Attempt to Negotiate Their Individual Process of Subjectification

The accounts of 18 young people with a history of NSSI and/or attempted suicide allowed us to elicit four superordinate themes which all speak about relationships: to the self, to others, to the body and senses, and finally to death. The experience of relationships is central to our participants' discourse—the relation to others and to the alter ego (perceived sometimes, as described above, as “another me”). Although the individual experience of construction of themselves (53) seems difficult for these adolescents with fragile narcissism, their action was always part of a relationship with another—real or fantasized. This is true for the NSSIs and for the suicide attempts. The process of separation-individuation is at the heart of the experience of these girls and young women and takes on different aspects that vary according to whether the youth's behavior involved NSSI, suicidal behavior, or both. The moments of self-attack can sometimes appear to be necessary to adolescents, enabling them to re-experience an undifferentiated state, but without settling permanently into it (71). The act sometimes came as an attempt to separate (as shown in the *Inner combat* theme), to break an attachment (as shown in *The act as a test of the separation process in adolescence* theme), sometimes as an effort at fusion (*The act: sending a message*) (46). Nonetheless, while NSSI can connect peers (72), suicide attempts seem instead to involve a process of subjectification.

The girls and young women, who seek to give a meaning to their distress, can see in their injuries a symbol of this distress. NSSI can become a banner of identity, the rallying sign of a group whose members recognize each other because they underwent the same—unspeakable—pain (73), as shown in the *NSSI that isolates and connects* theme. Nonetheless, for many this act is intimate, private; its traces must stay hidden from the eyes of others, to avoid attracting attention. This signature seems to respond to their need for a link between their apparently paradoxical desires—for both singularity and for a peer group—inherent in adolescence. NSSI comes to symbolize by this act the adolescent process between individuation and new connections. Le Breton (74) underlines the social logic of the act, which aims at passing through the thorny period of adolescence. From a sociological perspective, NSSI can be read as a necessary act of passage (74). We suggest that the individual fragility of these young people is influenced by the cultural (in the sense of societal) conditioning of modes of expressing distress.

It seems to be otherwise for the suicidal acts, described from the first-person singular perspective (“I”) in our results. The narrative changes from “we cut” to “I tried to kill myself.” That is, the suicidal act, although closely linked in many aspects to NSSI behavior, does not appear to take on this function of connection. Attempting suicide is perhaps more intimate, more personal, more desperate. Suicide attempts call out, but in a less interactive language, closing off dialogue or dumbfounding it. The mode of communication is not the same as in NSSI, for corporal labeling is not at the center of the process. The part of the figurative—visual—appeal of NSSI appears to be absent from suicide attempts (75). Moreover, there are fewer contagious phenomena in suicidal behavior. There is therefore a form of subjectification in suicide attempts, because its function is less connected, less identity-related.

The last level of our results concerns the relations that these girls and young women have with death (as shown in the *Relation to death* superordinate theme). We know that the idea of death is part of adolescence. It is consubstantial with the work of subjectification which leads individuals to think of themselves as such, individuals different from others, and to think about their thoughts, that is, to think about the meaning of their thoughts (76).

The act may not allow the symbolic elaboration of death. Some even consider that it expresses an inability to think of death (77). But at the same time, it can be seen as a symbolic ordering. The intentional injury allows a symbolic game with death and in this has a structuring function (as shown in the *Testing the lines between life and death* theme) (73, 78). Self-injurious behavior represents an attempt to control what these youth fear to undergo (79).

Our results bring to the fore the staging around the act (*Acting, alone on stage*). The body would thus constitute the stage on which the traces of a distress that cannot be represented are transposed (80). Accordingly, in this brush with death, these girls and young women stage what they are trying to symbolize psychologically (80), that is, “the wound that separates two edges,” the separation between self and other, between life and death. The use of staging appears to allow them to begin the work of subjectification, which has not been successfully accomplished in the mind and which must therefore be exteriorized. The girls can play different roles simultaneously: in cutting, for example, the victim passively watches herself bleed, the executioner who acts by cutting, and the spectator observing the spectacle of a mutilated body being viewed (81).

The psychopathological perspective teaches us that fragile adolescents have difficulty with subjectification. This may be explained by psychological symbolization activity—and therefore linkage activity—that is less effective or insufficiently invested, while the process of puberty is highly demanding of psychological functioning and all of its mechanisms of defense and adaptation (76). The recourse to artifice and to some form of unreality may be linked to this individual inability to represent and to the loss of meaning that can thus result.

Moreover, as anthropology teaches us, all societies are concerned with organizing the passage from childhood to adulthood. In some traditional societies, these rites of passage are more explicitly materialized. They envision these rites in bestowing efficacy on them: “*Without ritual, no symbolization of the passage”* (82). Initiation rites communicate to adolescents their status as adults and authenticate it (83). They mark the development of the construction of identity, recognized by the entire group. They are predetermined and codified by the adults to situate the novices, transmit the group's values and beliefs to them, record their membership and integrate them as active members of the community (84). Accordingly, the group can take charge of the work of subjectification in adolescence. The function of rituals is to organize and support transitions, which are sources of disorder (85).

In this respect, recourse to a form of scenography in the act appears very similar to a rite of passage. By rite or ritual, we mean a staging instituted with a symbolic meaning, following a specific order and leading to repeated behaviors (86). Reflecting our society's lack of symbolic and collective narrative around adolescence, these girls and young women embrace these acts in an attempt to negotiate their individual process of subjectification, neglecting to the extreme danger to which they are exposed.

## Limitations

We chose to explore the experience of adolescent girls. Because the study included no young boys, we were unable to examine the differences that might exist between girls and boys in their NSSI and suicidal acts. Several studies (87–89) have looked at gender differences in NSSI behavior and found similarities in terms of the age they start, the body sites chosen, and the association of suicidal ideation (89). Nonetheless, boys appear to use, besides NSSI, more diverse types of direct or indirect self-harming behaviors, such as substance abuse (90) or violence directed at others or at things, unlike girls who appear to use NSSI more exclusively. Moreover, the functions attributed to NSSI appear to differ by gender. Use of self-injury as an emotional regulator is shown less often in boys (89, 91). The epidemiologic data indicate that young adolescent girls attempt suicide more often but also that this gender difference attenuates with age (1, 66, 92, 93). One prospect for research is to continue this work among boys to detail these elements.

Because of the important theoretical debate on the question of the potential dichotomy between NSSI and suicidal behavior and due to our decision to use the qualitative process to avoid inducing the results of the theoretical or a priori assumptions, we decided not to differentiate the groups with NSSI and with SA. Subsequent studies may focus on one or the other group to examine the specificities of each experience in detail.

## Implications for Practice

Our results underline the relational aspects of the self-harming behavior (NSSI or suicidal) among adolescent girls and young women. These aspects also appear to be expressed in the family sphere, the social sphere, in contact with peers, and also at a societal level when the community no longer manages adolescence. When the process of subjectification does not appear to come to completion, these girls find themselves constrained to attack themselves. This observation allows us to consider the interest of using a systemic approach in the treatment of NSSI and suicidal acts (94). The involvement of the family or at least the parents so that they can be more active in treatment and can develop their own resources to help their child is essential. Moreover, the interest of involving the family in the care of young suicide attempters is widely recognized and in very diverse theoretical environments (95, 96). Although the systemic approach cannot be applied in all the situations encountered, it may nonetheless be useful to transpose some of its techniques to improve care for NSSI and attempted suicide. Family-focused interventions based especially on mentalization appear pertinent in view of our results (97). Organizing repeated family interviews where all family members are involved can be the occasion for the co-construction of meaning around the act. Work in a family framework on intrafamily communication and especially on how emotions are transmitted and named in the family might help adolescents to better mentalize their emotions and thus avoid the need for action (98, 99). More generally, allowing themselves to think that the symptom, like the skills and resources of treatment, belong to the entire family might let young people find a way out of the relational impasse in which they are very often trapped (49). These issues could be addressed in further qualitative studies.

## Ethics Statement

This study was carried out in accordance with the recommendations of an appropriate ethics review board (CEERB Paris Nord, IRB 00006477) with written informed consent from all subjects. All subjects (adolescents and their parents) gave written informed consent in accordance with the Declaration of Helsinki. The protocol was approved by the ethics review board CEERB Paris Nord.

## Author Contributions

SG, JL, and SS contributed conception and design of the study. SG and SS conducted the interviews. SG and JL analyzed the interviews to optimize the validity of the results. SG wrote the first draft of the manuscript. SG, SS, MS, JL, and MM wrote sections of the manuscript. All authors contributed to manuscript revision and read and approved the submitted version.

### Conflict of Interest Statement

The authors declare that the research was conducted in the absence of any commercial or financial relationships that could be construed as a potential conflict of interest.
